# Concomitant use of pembrolizumab and entinostat in adult patients with metastatic uveal melanoma (PEMDAC study): protocol for a multicenter phase II open label study

**DOI:** 10.1186/s12885-019-5623-3

**Published:** 2019-05-02

**Authors:** Henrik Jespersen, Roger Olofsson Bagge, Gustav Ullenhag, Ana Carneiro, Hildur Helgadottir, Ingrid Ljuslinder, Max Levin, Charlotta All-Eriksson, Bengt Andersson, Ulrika Stierner, Lisa M. Nilsson, Jonas A. Nilsson, Lars Ny

**Affiliations:** 1 Department of Oncology, Institute of Clinical Sciences, Sahlgrenska Academy at University of Gothenburg, Sahlgrenska University Hospital, Blå stråket 2, 413 45 Gothenburg, Sweden; 2 Department of Surgery, Institute of Clinical Sciences, Sahlgrenska Academy at University of Gothenburg, Sahlgrenska University Hospital, Blå stråket 5, 413 45 Gothenburg, Sweden; 3 Department of Radiology, Oncology and Radiation Science, Section of Oncology, Uppsala University, 751 05 Uppsala, Sweden; 4 Department of Oncology, Skåne University Hospital, Getingevägen 4, 221 85 Lund, Sweden; 5 Department of Oncology, Karolinska University Hospital, Karolinska vägen, 171 76 Stockholm, Sweden; 6 Department of Oncology, Norrlands University Hospital, 901 85 Umeå, Sweden; 7 St. Erik Eye Hospital, Polhemsgatan 50, 112 30 Stockholm, Sweden; 8 Department of Clinical immunology and transfusion medicine, Sahlgrenska University Hospital, Guldhedsgatan 10, 413 45 Gothenburg, Sweden; 9 Sahlgrenska Cancer Center, Sahlgrenska Academy at University of Gothenburg, Sahlgrenska University Hospital, Medicinaregatan 1F, 405 30 Gothenburg, Sweden

**Keywords:** Uveal melanoma, Metastatic, Immunotherapy, Programmed cell death 1 receptor, Pembrolizumab, Epigenetics, Histone deacetylase inhibitors, Entinostat

## Abstract

**Background:**

While recent years have seen a revolution in the treatment of metastatic cutaneous melanoma, no treatment has yet been able to demonstrate any prolonged survival in metastatic uveal melanoma. Thus, metastatic uveal melanoma remains a disease with an urgent unmet medical need. Reports of treatment with immune checkpoint inhibitors have thus far been disappointing. Based on animal experiments, it is reasonable to hypothesize that the effect of immunotherapy may be augmented by epigenetic therapy. Proposed mechanisms include enhanced expression of HLA class I and cancer antigens on cancer cells, as well as suppression of myeloid suppressor cells.

**Methods:**

The PEMDAC study is a multicenter, open label phase II study assessing the efficacy of concomitant use of the PD1 inhibitor pembrolizumab and the class I HDAC inhibitor entinostat in adult patients with metastatic uveal melanoma. Primary endpoint is objective response rate. Eligible patients have histologically confirmed metastatic uveal melanoma, ECOG performance status 0–1, measurable disease as per RECIST 1.1 and may have received any number of prior therapies, with the exception of anticancer immunotherapy. Twenty nine patients will be enrolled. Patients receive pembrolizumab 200 mg intravenously every third week in combination with entinostat 5 mg orally once weekly. Treatment will continue until progression of disease or intolerable toxicity or for a maximum of 24 months.

**Discussion:**

The PEMDAC study is the first trial to assess whether the addition of an HDAC inhibitor to anti-PD1 therapy can yield objective anti-tumoral responses in metastatic UM.

**Trial registration:**

ClinicalTrials.gov registration number: NCT02697630. (Registered 3 March 2016). EudraCT registration number: 2016–002114-50.

## Background

Uveal malignant melanoma (UM) is a disease that differs biologically from cutaneous melanoma. Uveal melanoma is a tumor in the eye arising from melanocytes in the uveal tract. It is most common in the choroidea (90%), but can also be found in the ciliary body (7%) and in the iris (3%) [1]. In Europe, the incidence shows a gradient from north-to-south, decreasing from over 8–9 per million in Scandinavia to less than 2 per million in the southern European countries [2, 3]. The incidence is increasing with age, and the median age at the time of diagnosis is about 60 years [4]. At the time of initial diagnosis, only 2–4% of all patients have metastatic disease [5] and many are cured from their primary tumor with initial surgical resection or radiotherapy of the eye. Very often the initial treatment results in low vision on the afflicted eye, reducing quality of life for these patients. Even worse, up to 50% of the patients will develop metastatic disease, most commonly with isolated liver metastases (89%), with a median survival of 6–12 months [5, 6]. Patients with metastases outside of the liver only, or when the liver is not the first site, seem to have a better survival [7]. For patients with liver metastases, regardless of treatment, the mortality rate is approximately 90% at 2 years with only about 1% of the patients surviving more than 5 years [6].

Currently explored treatment strategies for metastatic UM include liver directed therapies, chemotherapy, targeted therapies and immunotherapy. For patients with isolated liver metastases, liver directed therapies such as radiofrequency ablation, isolated hepatic perfusion (IHP) or percutan isolated hepatic perfusion (PHP), can induce objective responses, but a clear survival benefit has not been shown in prospective studies, although trials are ongoing [8].

The predominant mutations present in uveal melanoma are in *GNAQ* or the mutually exclusive *GNA11* gene, which are mutated in 90% of uveal melanomas. In the remaining 10%, recurrent mutations can be seen in *CYSLTR2* and *PLCB4* [9, 10]. *GNAQ/11* mutations result in activation of the Hippo and MAP-kinase pathways [11, 12]. Although the pathways have been delineated, a targeted therapy against GNAQ/GNA11 oncogenic driver mutations is lacking. Instead, attempts to target the downstream signaling pathways through inhibition of e.g. MEK or PKC have thus been tested, but not yet demonstrated clinical efficacy [13]. Another common genetic alteration in UM is inactivation or loss of the *BAP1* tumor suppressor gene, which results in metastatic progression [14].

Results of checkpoint blockade in patients with metastatic UM have so far been disappointing in the limited number of patients reported [15]. There are indeed scientific concerns raised about the feasibility of therapies such as those targeting Cytotoxic T-Lymphocyte Associated Protein 4 (CTLA-4) and/or Programmed cell Death protein 1 (PD-1). The eye is an immune privileged site and it is well known that primary uveal melanoma often has a reduced HLA class I expression. Low HLA expression can trigger NK cell lysis and high expression of HLA in the primary site is associated with worse prognosis [16]. If the low HLA expression, hampering recognition of cytotoxic T-cells and effective immunotherapies, is conserved in metastases has not been sufficiently studied. Unfortunately, there are no accurate animal models of GNAQ/11 mutated uveal melanoma which develop liver metastases, and biopsies from metastases, e.g. from the liver, have not been adequately characterized. Beyond checkpoint inhibition, other strategies for immunotherapy in UM include IMCgp100, a bispecific biological drug showing promising activity in early phase studies [17], and adoptive cell therapy with tumor infiltrating lymphocytes [18].

There have been several clinical trials with different kinds of chemotherapy, targeted therapies, immunotherapies, and liver directed therapies. Unfortunately, the median survivals reported in these trials do not differ from the anticipated survival of 6–12 months in patients not receiving antitumoral therapy [19]. Thus, there are strong arguments to investigate if anti-PD1 therapy can be effective in metastatic UM. However, the poor immunogenicity of uveal melanoma may interfere with the efficacy of anti-PD1 therapy, and published case series with PD1-inhibitors in monotherapy show very low response rates [15]. There is however emerging preclinical data indicating that the effect of immunotherapy may be augmented by epigenetic therapy [20]. For instance histone deacetylase (HDAC) inhibitors have been shown to **a)** enhance expression of HLA class I on cancer cells [21], **b)** trigger cell death recruiting immune cells [22], **c)** trigger DNA damage of uveal melanoma cells resulting in activation of danger signals [23], **d)** block myeloid-derived suppressor cell (MDSC) activity [24], and **e)** enhance the expression of cancer antigens silenced by immunoediting [25]. On the other hand, HDAC inhibitors also induce PD-L1 [26]. Therefore, addition of a PD-1/PD-L1 checkpoint inhibitor to HDAC inhibitors enhance the effect compared to monotherapy in vitro and in syngenic animal models [26, 27]. In particular, the HDAC inhibitor entinostat (MS-275) was demonstrated to enable durable responses to immune checkpoint inhibitors even when mice carried large tumors [24]. Given the poor responses to PD1 inhibitors in patients with UM, we hypothesize that an epigenetic therapy using entinostat could enhance the effect of PD1 inhibitor pembrolizumab in human patients.

Little is known about the toxicity of combined HDAC- and PD1-inhibition. In monotherapy, entinostat gives rise to manageable hematological and non-hematological toxicities, few of which are overlapping with those associated with PD1-inhibitors [28]. The dosing is based on results from the dose escalation cohort of an ongoing phase Ib/II trial of entinostat and pembrolizumab in other solid tumors (NCT02437136). According to interim data from the cutaneous melanoma expansion cohort of the same study, there are no obvious signs of synergistic toxic effects [29].

## Methods

### Study design

The PEMDAC study is a prospective multicenter, non-randomized, open label study, in which patients with stage IV UM are concomitantly treated with pembrolizumab 200 mg administered intravenously every third week and entinostat 5 mg administered orally once weekly.

### Endpoints

The primary endpoint is objective response rate (ORR) according to RECIST 1.1 criteria [30].

#### Secondary endpoints include

- Clinical benefit rate (CBR) (as of RECIST version 1.1 criteria): stable disease (SD) at 18 weeks OR any objective response
- Progression free survival (PFS)
- Overall survival (OS)
- Best overall response (BOR)
- Time to response (TTR)
- Duration of objective response (DOR)
- Incidence and severity of adverse events (AEs) and serious adverse events (SAEs)
- Eastern Cooperative Oncology Group (ECOG) Performance status (PS). Change from baseline in the ECOG PS at 18 weeks
- Changes in Quality of Life (QoL)

#### Exploratory endpoints include

- Radiological response (ORR, PFS) by immune related RECIST 1.1 criteria (irRECIST) [31]
- ORR in association with biomarker expression
- ORR in subgroups determined by to PD-L1 expression, serum LDH level, GNAQ/GNA11 mutation status and presence of extra-hepatic metastases
- Changes in number of myeloid-derived suppressor cells (MDSCs) and T-cell differentiation in peripheral blood and tumor biopsies pre- and post-therapy
- Changes in HLA expression in tumor biopsies pre- and post-therapy
- Changes in expression of immune checkpoint proteins in tumor biopsies pre- and post-therapy
- Changes in molecular characteristics of uveal melanoma metastases when treated with entinostat and pembrolizumab

### Study population

The investigated cohort of patients includes both untreated and previously treated patients.

#### Key inclusion criteria include

- Age above 18 years
- Signed and dated written informed consent before the start of specific protocol procedures
- ECOG PS 0–1
- Histologically/cytologically confirmed stage IV UM
- Measurable disease by computed tomography (CT) or Magnetic Resonance Imaging (MRI) per RECIST 1.1 criteria
- Any number of prior therapies (including none), with the exception of anticancer immunotherapy

#### Key exclusion criteria include

- Active brain metastases (symptomatic and/or requiring corticosteroids) or leptomeningeal metastases
- Previous treatment with anticancer immunotherapy
- Pregnant or nursing (lactating) women
- Medical, psychiatric, cognitive or other conditions that may compromise the patient’s ability to understand the patient information, give informed consent, comply with the study protocol or complete the study
- Active autoimmune disease
- Immune deficiency or treatment with systemic corticosteroids (> 10 mg daily prednisone equivalents).
- Use of other investigational drugs (drugs not marketed for any indication) within 4 weeks before study drug administration
- Life expectancy of less than 3 months

### Treatments and evaluation

**Fig. 1:**
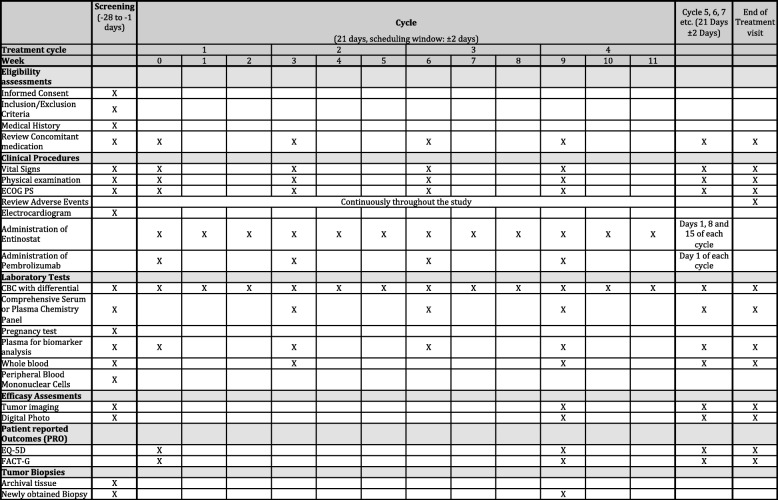
Schedule of enrolment, interventions and assessments

A schedule of enrolment, interventions and assessments is shown in Fig. 1.

### Dosing and administration

Entinostat is to be taken on an empty stomach, at least 2 h after a meal and at least 1 h before the next meal. If an entinostat dose is missed, it may be taken up to 48 h after the scheduled dosing time. If it is not taken within the 48-h window, the dose should not be taken and should be counted as a missed dose. The patient should take the next scheduled dose per protocol. If entinostat is vomited, dosing should not be re-administered but instead the dose should be skipped.

### Efficacy assessments

The subjects will be followed for 2 years. Study visits will be carried out at baseline, and week 3, 6, 9, 12, 15, 18, 21 and every 3 weeks until 24 months.

- MRI or CT (as preferred by the investigator) of chest and abdomen will be carried out every 9 weeks
- EQ5D Quality of Life questionnaire at scheduled visit baseline and then every 9 weeks until 24 months
- Blood samples of biomarkers will be collected at every scheduled visit

### Safety and tolerability

The subjects will be followed for 2 years. Study visits will be carried out at baseline, and week 3, 6, 9, 12, 15, 18, 21 and every 3 weeks until 24 months. All AEs and SAEs will be collected. At all visits the following tests will be performed: Blood samples (Hgb, WBC, Plt, INR, APTT, AST, ALT, ALP, Bilirubin, LDH, TSH, free-T4/free-T3, Creatinine and electrolytes).

### Duration

Study treatment will be continued until documented disease progression, intolerable side effects, patient’s withdrawal of consent or decision of the investigating physician to end treatment, or to a maximum of 2 years. The subject is free to withdraw from the study for any reason and at any time without giving reason for doing so and without penalty or prejudice. The investigator is also free to terminate a subject’s study treatment at any time if the subject’s clinical condition warrants it.

Discontinuation Criteria for Individual Subjects:

1. Inappropriate enrollment (violation of Inclusion/Exclusion criteria).
2. Withdrawal of consent.
3. Progression of disease.
4. Discontinuation of study drugs due to AE.

### Treatment beyond radiological progression

Due to the unique tumor response characteristics seen with treatment with immunotherapy, standard RECIST 1.1 may not provide an accurate response assessment. Modifications to RECIST 1.1 Criteria (irRECIST) takes into account these patterns of atypical response and enable treatment beyond initial radiographic progression. IrRECIST will therefore be used for assessment of tumor response for managing subjects on protocol treatment and decision making for discontinuation of study therapy due to disease progression. If imaging shows PD, it is at the discretion of the investigator to keep a patient on study treatment or to stop study treatment until imaging is repeated approximately 4 weeks later in order to confirm PD, as described in the irRECIST recommendations [31]. Patients that are deemed clinically unstable or who have biopsy proven new metastatic lesions are not required to have repeat imaging for confirmation. This decision will be based on clinical judgment of a patient’s overall clinical condition, including performance status, clinical symptoms, and laboratory data. At a minimum, patients must meet the following criteria for continued treatment on study after disease progression is identified at a tumor assessment:

- Absence of new or worsening symptoms
- No decline in ECOG performance status
- Absence of rapid progression of disease or of
- Absence of progressive tumor at critical anatomical sites (e.g., cord compression) requiring urgent alternative medical intervention

### Statistical considerations

The sample size and power estimation is based on the primary endpoint ORR, only. Power is required to be 80%, Significance is generally set to 5%. We assume that an ORR of 5% is not a clinically relevant treatment effect, whereas 20% is sufficient to consider the treatment useful. Enrollment will continue until the required sample size has been reached. Patients will be enrolled in two batches, first consisting of 10 patients and the second group of 19. The second sample will not be recruited if the result from the 10 first is considered inadequate. This is the optimal allocation according to Simon’s Optimal Two Stage Design (significance level = 5% (one-sided)) [32]. If no objective responses (CR or PR) are reported after the first stage of 10 patients, the study is interrupted early for futility. The expected number of patients is estimated to 17.6. Outcome measures that are proportions will be reported using a 95% confidence interval. Since the sample size is small an exact method will be used. If applicable, tests are conducted versus zero or highest non-efficient value.

The primary and secondary outcomes can be divided into the following groups:

- Proportions of successful events: ORR, CBR, BOR, Incidence of AE, ECOG PS
- Time to event: OS, PFS, TTR, DOR
- Others: QoL difference to baseline

#### Analysis of proportions

Outcome measures that are proportions will be reported using a 95% confidence interval. Since the sample size is small an exact method will be used. If applicable, tests are conducted versus zero or highest non-efficient value.

#### Analysis of time to event

Outcome measures that are times to various events will be analyzed using non-parametric methods. Time is summarized using medians, together with 95% confidence intervals. If applicable, test is conducted versus zero or highest non-efficient value. Results are graphically presented using Kaplan-Meier survival curves.

#### Quality of life (QoL) difference from baseline

QoL difference will be analyzed using non-parametric methods, e.g. Mann-Whitney-Wilcoxon test.

## Discussion

Metastatic UM represents a major unmet medical need in oncology. Being a rare disease and of low commercial value, drug discovery in UM is of lower priority by the pharmaceutical industry. The PEMDAC study is the first trial to assess whether the addition of an HDAC inhibitor to anti-PD1 therapy can yield objective anti-tumoral responses in metastatic UM. The study is investigator initiated, is carried out at the major Swedish University hospitals (with the possibility of referrals from other community and academic hospitals) and is coordinated by the Swedish Melanoma Study Group (SMSG).

One aim of the PEMDAC trial is to include a study population representative of that seen in the clinic, thus applying generous eligibility criteria including the allowance for any number of previous therapies. This approach risks generating a heterogeneous study population, but is in our view justified by the urgent medical need in metastatic UM and the prospect to include a sufficient number of patients during a reasonable recruitment period.

All investigators in the trial have substantial experience in the management of immune related adverse events associated with immunotherapy.

The Committee for Medicinal Products for Human Use (CHMP)/European Medicines Agency (EMA) has in 2017 presented “Guideline on the evaluation of anticancer medicinal products in man” and suggests ORR to be used as primary endpoint in exploratory single armed studies as in the present study. However, since the conception and approval of PEMDAC, there is data suggesting that ORR might be challenged as the most appropriate primary endpoint for phase II studies of immunotherapy in cancer, as it risks underestimating the treatment benefit. A retrospective analysis of phase II immunotherapy trials in cancer showed that PFS at 6 months might be a better correlate for OS in this setting [33]. Furthermore, modified RECIST criteria (irRECIST) have been developed to account for the unconventional response characteristics associated with immunotherapies [31]. Progression free survival (including landmark analysis) and ORR according to irRECIST will be analysed as secondary endpoints. However, these alternative surrogate endpoints have not yet been validated in prospective studies across tumor types. Furthermore, pseudoprogression appears to be uncommon using PD1-inhibitors, and the benefit of durable conventional objective response remains undisputed. We believe that the present study design is appropriate to investigate whether there are observations of clinically relevant efficacy from combined HDAC- and PD1-inhibition in UM patients, to warrant further investigation in larger cohorts.

With 29 patients, the present study represents a significant cohort of patients with this very rare disease. An important aim of the study is therefore explorative analyses, with the aim of increasing our understanding of the immunological and genetic nature of UM, and possibly to detect markers predictive for response.

### Trial status

The trial started enrolment of patients in February 2018, and recruited patients are in active treatment phase or follow-up. An updated trial status will be continuously displayed at Clinicaltrials.gov. The primary endpoint will be assessed 6 months after last patient first visit. Key efficacy and safety results will be reported thereafter, preliminary late 2019. Latest protocol version and date: 1.4, 2018-06-29.

## Abbreviations

AE: Adverse event
BOR: Best overall response
CBR: Clinical benefit rate
CT: Computed tomography
CTLA-4: Cytotoxic T-lymphocyte associated protein 4
DOR: Duration of objective response
ECOG: Eastern Cooperative Oncology Group
HDAC: Histone deacetylase
IHP: Isolated hepatic perfusion
irRECIST: Immune related RECIST 1.1 criteria
MDSC: Myeloid-derived suppressor cell
MRI: Magnetic Resonance Imaging
ORR: Objective response rate
OS: Overall Survival
PD-1: Programmed cell death protein 1
PFS: Progression free survival
PS: Performance status
QoL: Quality of life
SAE: Serious adverse event
SD: Stable disease
SMSG: Swedish melanoma study group
TTR: Time to response
UM: Uveal malignant melanoma

## References

[1] Damato B (2006). Treatment of primary intraocular melanoma. Expert Rev Anticancer Ther.

[2] Bergman L, Seregard S, Nilsson B, Ringborg U, Lundell G, Ragnarsson-Olding B (2002). Incidence of uveal melanoma in Sweden from 1960 to 1998. Invest Ophthalmol Vis Sci.

[3] Virgili G, Gatta G, Ciccolallo L, Capocaccia R, Biggeri A, Crocetti E, Lutz J-MM, Paci E, group E (2007). Incidence of uveal melanoma in Europe. Ophthalmology.

[4] Eskelin S (2002). Mode of presentation and time to treatment of uveal melanoma in Finland. British Journal of Ophthalmology.

[5] Kujala E, Makitie T, Kivela T (2003). Very long-term prognosis of patients with malignant uveal melanoma. Invest Ophthalmol Vis Sci.

[6] Diener-West M, Reynolds SM, Agugliaro DJ, Caldwell R, Cumming K, Earle JD, Hawkins BS, Hayman JA, Jaiyesimi I, Jampol LM (2005). Development of metastatic disease after enrollment in the COMS trials for treatment of choroidal melanoma: collaborative ocular melanoma study group report no. 26. Arch Ophthalmol.

[7] Kath R, Hayungs J, Bornfeld N, Sauerwein W, Höffken K, Seeber S (1993). Prognosis and treatment of disseminated uveal melanoma. Cancer.

[8] Olofsson R, Ny L, Eilard MS, Rizell M, Cahlin C, Stierner U, Lonn U, Hansson J, Ljuslinder I, Lundgren L (2014). Isolated hepatic perfusion as a treatment for uveal melanoma liver metastases (the SCANDIUM trial): study protocol for a randomized controlled trial. Trials.

[9] Moore AR, Ceraudo E, Sher JJ, Guan Y, Shoushtari AN, Chang MT, Zhang JQ, Walczak EG, Kazmi MA, Taylor BS (2016). Recurrent activating mutations of G-protein-coupled receptor CYSLTR2 in uveal melanoma. Nat Genet.

[10] Johansson P, Aoude LG, Wadt K, Glasson WJ, Warrier SK, Hewitt AW, Kiilgaard JF, Heegaard S, Isaacs T, Franchina M (2016). Deep sequencing of uveal melanoma identifies a recurrent mutation in PLCB4. Oncotarget.

[11] Van Raamsdonk CD, Bezrookove V, Green G, Bauer J, Gaugler L, O’Brien JM, Simpson EM, Barsh GS, Bastian BC (2009). Frequent somatic mutations of GNAQ in uveal melanoma and blue naevi. Nature.

[12] Van Raamsdonk CD, Griewank KG, Crosby MB, Garrido MC, Vemula S, Wiesner T, Obenauf AC, Wackernagel W, Green G, Bouvier N (2010). Mutations in GNA11 in uveal melanoma. N Engl J Med.

[13] Carvajal RD, Piperno-Neumann S, Kapiteijn E, Chapman PB, Frank S, Joshua AM, Piulats JM, Wolter P, Cocquyt V, Chmielowski B (2018). Selumetinib in combination with Dacarbazine in patients with metastatic uveal melanoma: a phase III, multicenter, randomized trial (SUMIT). J Clin Oncol.

[14] Harbour JW, Onken MD, Roberson ED, Duan S, Cao L, Worley LA, Matatall KA, Helms C, Bowcock AM, Council ML (2010). Frequent mutation of BAP1 in metastasizing uveal melanomas. Science.

[15] Algazi AP, Tsai KK, Shoushtari AN, Munhoz RR, Eroglu Z, Piulats JM, Ott PA, Johnson DB, Hwang J, Daud AI (2016). Clinical outcomes in metastatic uveal melanoma treated with PD-1 and PD-1L antibodies. Cancer.

[16] Ericsson C, Seregard S, Bartolazzi A, Levitskaya E, Ferrone S, Kiessling R, Larsson O (2001). Association of HLA class I and class II antigen expression and mortality in uveal melanoma. Invest Ophthalmol Vis Sci.

[17] Sato Takami, Nathan Paul D., Hernandez-Aya Leonel Fernando, Sacco Joseph J, Orloff Marlana M., Truscello Jessica, McAlpine Cheryl, Hulstine Ann-Marie, Lanasa Mark C., Coughlin Christina Marie, Carvajal Richard D. (2017). Intra-patient escalation dosing strategy with IMCgp100 results in mitigation of T-cell based toxicity and preliminary efficacy in advanced uveal melanoma. Journal of Clinical Oncology.

[18] Chandran SS, Somerville RP, Yang JC, Sherry RM, Klebanoff CA, Goff SL, Wunderlich JR, Danforth DN, Zlott D, Paria BC (2017). Treatment of metastatic uveal melanoma with adoptive transfer of tumour-infiltrating lymphocytes: a single-Centre, two-stage, single-arm, phase 2 study. Lancet Oncol.

[19] Yang J, Manson DK, Marr BP, Carvajal RD (2018). Treatment of uveal melanoma: where are we now?. Ther adv med oncol.

[20] Weintraub K (2016). Take two: combining immunotherapy with epigenetic drugs to tackle cancer. Nat Med.

[21] Campoli M, Ferrone S (2008). HLA antigen changes in malignant cells: epigenetic mechanisms and biologic significance. Oncogene.

[22] Landreville S, Agapova OA, Matatall KA, Kneass ZT, Onken MD, Lee RS, Bowcock AM, Harbour WJ (2012). Histone deacetylase inhibitors induce growth arrest and differentiation in uveal melanoma. Clin Cancer Res.

[23] Lee JH, Choy ML, Ngo L, Foster SS, Marks PA (2010). Histone deacetylase inhibitor induces DNA damage, which normal but not transformed cells can repair. Proc Natl Acad Sci U S A.

[24] Kim K, Skora AD, Li Z, Liu Q, Tam AJ, Blosser RL, Diaz LA, Papadopoulos N, Kinzler KW, Vogelstein B, Zhou S (2014). Eradication of metastatic mouse cancers resistant to immune checkpoint blockade by suppression of myeloid-derived cells. Proc Natl Acad Sci.

[25] Maio M, Coral S, Fratta E, Altomonte M, Sigalotti L (2003). Epigenetic targets for immune intervention in human malignancies. Oncogene.

[26] Woods DM, Sodre AL, Villagra A, Sarnaik A, Sotomayor EM, Weber J (2015). HDAC inhibition upregulates PD-1 ligands in melanoma and augments immunotherapy with PD-1 blockade. Cancer Immunol Res.

[27] Booth L, Roberts JL, Poklepovic A, Kirkwood J, Dent P (2017). HDAC inhibitors enhance the immunotherapy response of melanoma cells. Oncotarget.

[28] Hauschild A, Trefzer U, Garbe C, Kaehler K, Ugurel S, Kiecker F, Eigentler T, Krissel H, Schadendorf D. A phase II multicenter study on the histone deacetylase (HDAC) inhibitor MS-275, comparing two dosage schedules in metastatic melanoma. J Clin Oncol. 2006;24 (abstr 8044).

[29] Agarwala Sanjiv S., Moschos Stergios J., Johnson Melissa Lynne, Opyrchal Mateusz, Gabrilovich Dmitry, Danaher Patrick, Wang Fang, Brouwer Susan, Ordentlich Peter, Sankoh Serap, Schmidt Emmett V., Meyers Michael L., Sullivan Ryan J. (2018). Efficacy and safety of entinostat (ENT) and pembrolizumab (PEMBRO) in patients with melanoma progressing on or after a PD-1/L1 blocking antibody. Journal of Clinical Oncology.

[30] Eisenhauer EA, Therasse P, Bogaerts J, Schwartz LH, Sargent D, Ford R, Dancey J, Arbuck S, Gwyther S, Mooney M (2009). New response evaluation criteria in solid tumours: revised RECIST guideline (version 1.1). Eur J Cancer.

[31] Nishino M, Giobbie-Hurder A, Gargano M, Suda M, Ramaiya NH, Hodi FS (2013). Developing a common language for tumor response to immunotherapy: immune-related response criteria using unidimensional measurements. Clin Cancer Res.

[32] Simon R (1989). Optimal two-stage designs for phase II clinical trials. Controlled clin trials.

[33] Ritchie G, Gasper H, Man J, Lord S, Marschner I, Friedlander M, Lee CK (2018). Defining the Most appropriate primary end point in phase 2 trials of immune checkpoint inhibitors for advanced solid cancers: a systematic review and meta-analysis. JAMA oncol.

